# Functional analysis of polyphenol oxidase 1 gene in common wheat

**DOI:** 10.3389/fpls.2023.1171839

**Published:** 2023-07-31

**Authors:** Shengnan Zhai, Hang Liu, Xianchun Xia, Haosheng Li, Xinyou Cao, Zhonghu He, Wujun Ma, Cheng Liu, Jianmin Song, Aifeng Liu, Jingjuan Zhang, Jianjun Liu

**Affiliations:** ^1^ National Engineering Laboratory for Wheat and Maize, Key Laboratory of Wheat Biology and Genetic Improvement in the Northern Yellow-Huai Rivers Valley of Ministry of Agriculture and Rural Affairs, Crop Research Institute, Shandong Academy of Agricultural Sciences, Jinan, China; ^2^ Australian-China Joint Centre for Wheat Improvement, Western Australian State Agriculture Biotechnology Centre, College of Science, Health, Engineering and Education, Murdoch University, Perth, WA, Australia; ^3^ National Wheat Improvement Center, Institute of Crop Sciences, Chinese Academy of Agricultural Sciences, Beijing, China

**Keywords:** gene function, genetic regulation, RNAi, PPO activity, TILLING

## Abstract

Polyphenol oxidase (PPO) activity is a major cause of the undesirable brown color of wheat-based products. *Ppo1*, a major gene for PPO activity, was cloned based on sequence homology in previous studies; however, its function and regulation mechanism remain unclear. In this study, the function and genetic regulation of *Ppo1* were analyzed using RNA interference (RNAi) and Targeting Induced Local Lesions IN Genomes (TILLING) technology, and superior mutants were identified. Compared with the control, the level of *Ppo1* transcript in RNAi transgenic lines was drastically decreased by 15.5%–60.9% during grain development, and PPO activity was significantly reduced by 12.9%–20.4%, confirming the role of *Ppo1* in PPO activity. Thirty-two *Ppo1* mutants were identified in the ethyl methanesulfonate (EMS)-mutagenized population, including eight missense mutations, 16 synonymous mutations, and eight intron mutations. The expression of *Ppo1* was reduced significantly by 6.7%–37.1% and 10.1%–54.4% in mutants M092141 (G311S) and M091098 (G299R), respectively, in which PPO activity was decreased by 29.7% and 28.8%, respectively, indicating that mutation sites of two mutants have important effects on PPO1 function. Sequence and structure analysis revealed that the two sites were highly conserved among 74 plant species, where the frequency of glycine was 94.6% and 100%, respectively, and adjacent to the entrance of the hydrophobic pocket of the active site. The M092141 and M091098 mutants can be used as important germplasms to develop wheat cultivars with low grain PPO activity. This study provided important insights into the molecular mechanism of *Ppo1* and the genetic improvement of wheat PPO activity.

## 1 Introduction

Polyphenol oxidase (PPO) catalyzes the conversion of phenolic compounds to quinones, which in turn react with amines and thiol groups or undergo self-polymerization to produce dark or brown melanins (Baik and Ullrich, 2008; Taranto et al., 2017). PPO activity in grain is a crucial factor in the undesirable time-dependent discoloration and darkening of wheat end-used products (Anderson and Morris, 2003; Fuerst et al., 2006; Parveen et al., 2010; Morris, 2018). The discoloration largely reduces the nutritional quality of wheat products and negatively affects consumer acceptance (Simeone et al., 2002; Akond et al., 2010; Martin et al., 2011). Therefore, developing wheat cultivars with low grain PPO activity is the best way to reduce undesirable darkening and has always been an important goal in wheat breeding.

The knowledge of the genetic control of PPO activity could enable the development of better strategies in wheat breeding programs to reduce undesirable darkening. To date, efforts have been made to identify the molecular mechanism underlying PPO activity in wheat (Zhang et al., 2005; Watanabe et al., 2006; Sadeque and Turner, 2010; Zhai et al., 2020). A number of quantitative trait loci (QTL) analyses indicated that genes located on homoeologous group 2 chromosomes are the most significant determinant of PPO activity in wheat grains (Demeke et al., 2001a; Raman et al., 2005; Zhai et al., 2016a). *Ppo1* mapped on homoeologous group 2 was cloned by homology-based cloning and co-localized with PPO activity; however, its function and genetic regulation remain unclear (Jukanti et al., 2006; He et al., 2007; Nilthong et al., 2012; Hystad et al., 2015).

RNA interference (RNAi) is a very powerful tool for the analysis of gene function, especially for common wheat where it can simultaneously silence all three homoeologs by a single RNAi construct (McGinnis, 2010; Liu et al., 2021). Until now, RNAi has been used to investigate the function of a wide range of genes in wheat, such as inositol pentakisphosphate kinase (*IPK1*), grain weight 2 (*GW2*), and nucleoredoxin (*NRX1*) genes (Aggarwal et al., 2018; Sestili et al., 2019; Zhang et al., 2021). Targeting Induced Local Lesions IN Genomes (TILLING) combining chemical mutagenesis with a high-throughput screen for mutations can generate a large number of allelic variations which provide valuable mutants to understand the role and genetic regulation of target genes (Stemple, 2004; Slade et al., 2005). Meanwhile, the obtained excellent mutants can be directly applied to breeding programs because they do not involve a transgenic operation and can stably be inherited (Slade et al., 2012; Chen et al., 2014).

The aims of the present study were to 1) confirm the function of the *Ppo1* gene on PPO activity in wheat grains by RNAi, 2) understand the role and genetic regulation of *Ppo1* by TILLING, and 3) finally provide superior germplasm resources for breeding wheat varieties with low PPO activity.

## 2 Materials and methods

### 2.1 Construction of transformation vectors

**Figure 1:**
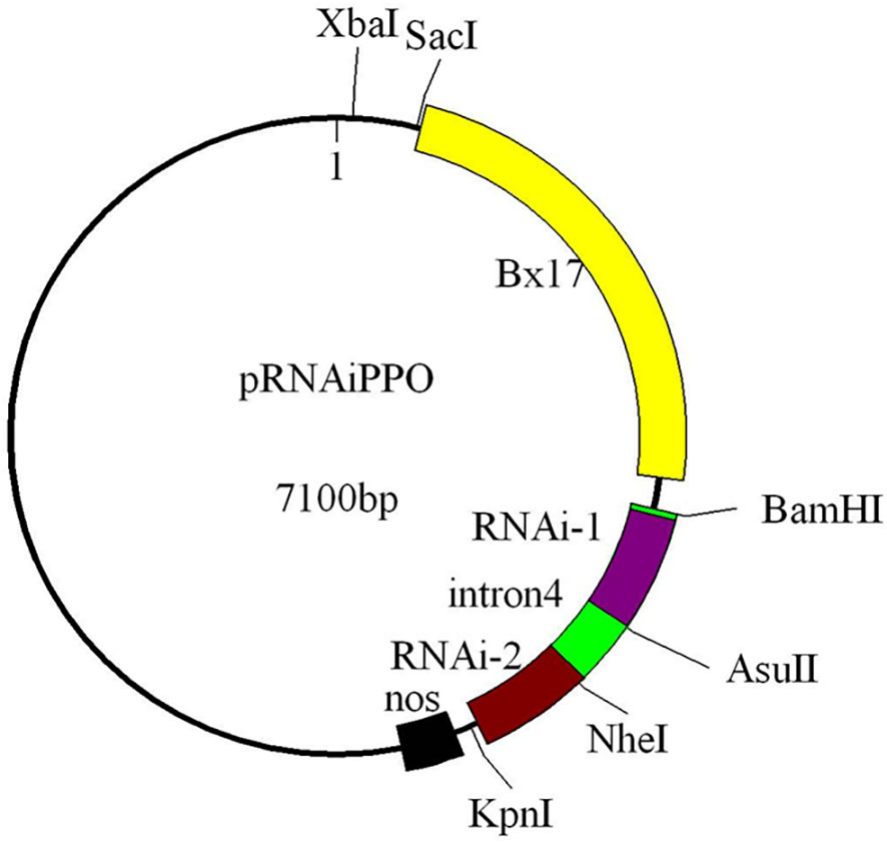
Diagram showing the RNAi cassette in the transformation plasmid (pRNAiPpo1). The trigger fragment of *Ppo1* is placed in the forward (RNA-1) and reverse (RNA-2) orientations separated by the fourth intron of the wheat *Psy1* gene (intron4). The restriction sites used in the RNAi vector construction are indicated. The diagram is not drawn in scale. Bx17, endosperm-specific promoter; nos, *Agrobacterium tumefaciens* nopaline synthase (*Nos*) terminator.

The RNAi construct was designed to evaluate the impact of *Ppo1* on grain PPO activity. Briefly, the binary vector pSAABx17, containing the endosperm-specific promoter of high molecular weight glutenin subunit (HMW-GS) gene *Bx17*, the nopaline synthase (*Nos*) terminator, and a selectable neomycin phosphotransferase II (*nptII*) gene conferring resistance to kanamycin, was used to construct an RNAi vector. The cDNA sequences of *Ppo-A1*, *Ppo-B1*, and *Ppo-D1* (GenBank accession nos. EF070147, EF070149, and GQ303713, respectively) were highly conserved, with 95.4% sequence identity among each other. All three *Ppo1* homoeologs possessed an open reading frame of 1,731 bp and contained three exons of 596, 262, and 873 bp, respectively. A part of the third exon of *Ppo1* (454 bp) was selected as the trigger fragment and amplified using specific primers with incorporated restriction sites (**Table S1**). The fourth intron of the wheat *Psy1* gene (GenBank accession no. EF600063) used as the spacer was amplified by primers *In-F* and *In-R* and ligated into the pSAABx17 vector. The sense and antisense fragments were inserted into the pSAABx17 plasmid with each one separated by the spacer, and all sequences and directions of the inserts were verified by sequencing (Sangon Biotech, Shanghai, China). The final RNAi construct was named pRNAiPPO1 (**Figure 1**).

### 2.2 Development of RNAi transgenic lines

The pRNAiPPO1 was transformed into wheat cultivar NB1 using the *Agrobacterium*-mediated method following Nadolska-Orczyk et al. (2005). The *Ppo1* genotypes of NB1 were examined by primers PPO18, F-8, PPO16, and PPO29 (Sun et al., 2005; He et al., 2007; Si et al., 2012), displaying *PPO-A1a*, *PPO-B1a*, and *PPO-D1a*, with high PPO activity. Regenerated plants were screened using kanamycin. Surviving plants were transferred to soil and grown to maturity under growth chamber conditions as described by Zhai et al. (2016b). Transformed plants were further confirmed by PCR analysis using two primer pairs: one specific for the *FAD2* intron, a part of the pRNAiPPO1 construct, and another for the trigger fragment of the *Ppo1* gene (**Table S1**). The PCR-positive transgenic plants were self-pollinated, and seeds from each plant were separately harvested in the following generations.

Non-segregant transgenic T_3_ lines and non-transformed controls were grown under field conditions in Jinan, Shandong Province, during the 2019–2020 cropping season. Seeds were sown in 3 m rows with 30 plants per row, 25 cm between rows, and 5 rows per transgenic line. Plants were verified by PCR and individually marked at anthesis for later collection. Grains were collected from five plants for the expression analysis of *Ppo1* genes and PPO activity assays. Briefly, immature seeds were collected 7, 14, 21, and 28 days post-anthesis (DPA), immediately frozen in liquid nitrogen, and stored at −80°C for RNA isolation. Mature seeds were harvested, milled by a Cyclotec™ 1093 mill (Foss Tecator Co., Hillerod, Denmark), and stored at −20°C for the measurement of PPO activity.

### 2.3 *Ppo1* expression analysis

Total RNA was extracted using the RNAprep Pure Plant Kit (Tiangen Biotech, Beijing, China) following the manufacturer’s instructions. RNA concentration and purity were determined by a NanoDrop ND-2000 spectrophotometer (Thermo Scientific, Wilmington, USA). cDNA was synthesized using a PrimeScript™ RT Reagent Kit (TaKaRa, Dalian, China) according to the manufacturer’s protocol.

Quantitative real-time PCR (qRT-PCR) was performed with a Roche LightCycler 480 (Roche Applied Science, Indianapolis, IN, USA) in a 20-µL reaction system containing 10 µL of LightCycler FastStart SYBR Green Master Mix (Roche Applied Science), 0.4 µM of each primer, 50 ng of cDNA, and ddH_2_O supplement to reach 20 μL. The cycling program was 95°C for 10 min, followed by 40 cycles of 95°C for 15 s, 60°C for 20 s, and 72°C for 20 s. Fluorescence was acquired at 60°C. The primers amplifying all three *Ppo1* genes were designed based on conserved regions among the A, B, and D subgenomes (**Table S2**). The specificity of amplification was determined by sequencing qRT-PCR products and melting curve analysis. The *Ppo1* transcript level was normalized to the expression of the *β-actin* gene (AB181991) as a constitutively expressed internal control. The formula 2^−ΔΔCT^ was used to calculate the relative expression of *Ppo1* genes (Livak and Schmittgen, 2001). First, the relative expression level of *Ppo1* genes was corrected by the *β-actin* gene transcription level in the same sample. Secondly, the relative expression of *Ppo1* genes of non-transformed control at 7 DPA was set as 1, and the relative expression of *Ppo1* genes of different transgenic lines at different grain development stages was calculated. For each transgenic line and non-transformed control, three biological replicates with three technical repeats each were performed, and data were shown as mean ± standard deviation.

### 2.4 PPO activity assay

PPO activity was determined using a modified protocol of AACC 22-85.01 (American Association of Cereal Chemists International, 2010). Briefly, 0.2 g of whole flour was incubated in 1.5 mL of 10 mM L-3,4-dihydroxyphenylalanine (L-DOPA) in 50 mM of 3-(N-morpholino)propanesulfonic acid (MOPS) buffer (pH 6.5) in a 50-mL centrifuge tube, constantly rotated on a reciprocating shaker for 30 min at 22°C. After centrifuging at 5,000 rpm for 10 min, the absorbance was measured at 475 nm with 200 μL of the supernatant using an Absorbance Microplate Reader (SpectraMax Plus 384, Molecular Devices, LLC, USA) and converted to PPO activity (U g^−1^ min^−1^). For each transgenic line and non-transformed control, five biological replicates with three technical repeats each were performed, and the mean values were used for statistical analyses. PPO activity of the non-transformed control was designated as the calibrator with its value set to 1.

### 2.5 Subcellular localization of PPO1 in wheat

To determine the subcellular localization of PPO1, the cDNA of *Ppo1* without the termination codon was amplified and C-terminally fused to the green fluorescent protein (GFP) gene in the pAN580 vector to create Ppo1-GFP under the control of the cauliflower mosaic virus (CaMV) 35S promoter. The primers were Ppo1-GFP-F (5′-GCCCAGATCAACTAGTATGGAGAGCAGTCGCATGCCAC-3′) and Ppo1-GFP-R (5′-T TCGAGACGTCTCTAGACTTGACGTAGCTGATGCTGACG-3′). The Ppo1-GFP fusion and GFP were transiently transformed into wheat protoplasts following the protocols of Zhai et al. (2016b). Fluorescence images were observed using a Zeiss LSM710 confocal laser microscope (Carl Zeiss MicroImaging GmbH, Germany).

### 2.6 Screening of *Ppo1* mutants by TILLING

Two EMS-mutagenized populations (Jimai 20: 1,251 M_2_ lines; Jimai 22: 1,240 M_2_ lines) were constructed and described in a previous study (Zhai et al., 2016b). The total genomic DNA of each M_2_ generation plant was extracted, and eight DNA samples were mixed equally to form a DNA pool and organized in 96-well plates. *Ppo1* mutants were screened by TILLING technology according to Till et al. (2006) with minor modifications. The procedures were as follows: 1) The *Ppo1* genotypes of Jimai 20 and Jimai 22 were examined as mentioned above and displayed *PPO-A1b*/*PPO-B1a/PPO-D1a* and *PPO-A1b*/*PPO-B1a*/*PPO-D1b*, respectively. According to sequence polymorphisms among *Ppo1* homologous genes, A, B, and D homolog-specific primers were designed for screening the mutant library (**Table S3**). The subgenomic specificity of the primers was verified based on Chinese Spring nulli-tetrasomic lines and sequencing of PCR products; 2) PCR amplification was carried out with DNA pool as a template, and the amplified products were repeatedly denatured and annealed to form a heteroduplex between wild type and mutant; 3) the heteroduplex was digested by the mismatch-specific endonuclease CEL I, and the products were separated by non-denatured polyacrylamide gel electrophoresis (PAGE) to screen DNA pools containing mutant sites; 4) each single plant DNA from the positive DNA pool was mixed with wild-type DNA and re-screened as described above to identify single mutant plants; and 5) the amplified product of the mutant plant was cloned and sequenced to obtain the variation information of *Ppo1* genes. All *Ppo1* mutants were deposited in the National Genebank of China (Chinese Academy of Agricultural Sciences) and available after approval.

### 2.7 Mutant effect detection

To analyze the effects of mutation sites on *Ppo1* gene expression and PPO activity, homozygous M_3_ plants with missense mutation sites were backcrossed with wild-type plants to construct F_2_ populations to reduce background noise. F_2_ populations were grown in Jinan during the 2020–2021 cropping season. Seeds were sown in 3 m rows with 30 plants per row, 25 cm between rows, and 10 rows per F_2_ population. Conventional field management was adopted.

The *Ppo1* genotype (homozygous mutant, heterozygous mutant, and wild type) of each line in F_2_ populations was identified by sequencing. In each F_2_ population, five plants of each genotype with the same growth and development process were selected and used for subsequent analysis. Briefly, the flowering period of each plant was tagged, and grains were collected from 7 to 28 DPA at 7-day intervals for RNA extraction and the method for *Ppo1* gene expression analysis was mentioned above. The relative expression levels of *Ppo1* were firstly normalized to the transcript level of the *β-actin* gene in the same sample and then calculated relative to the value of wild-type genotypes at 7 DPA (set to 1) in each F_2_ population. The mature grains were harvested per plant for determination of PPO activity following the method described above. The PPO activity of wild-type genotypes in each F_2_ population was designated as the calibrator with its value set to 1. The impacts of new *Ppo1* alleles on gene expression and PPO activity were assessed by comparing the differences between homozygous or heterozygous mutants and wild-type genotypes in each F_2_ population.

### 2.8 Prediction of the functional domain and structure model of PPO1

Based on the Phytozome v13 database (http://www.phytozome-next.jgi.doe.gov/), the amino acid sequences of *Ppo1* genes from 74 species were obtained (**Table S4**). The functional domains of PPO1 proteins were predicted by the National Center for Biotechnology Information Conserved Domain Database (NCBI’CCD, http://www.ncbi.nlm.nih.gov/cdd) and MEME Suite v5.1.0 (http://meme-suite.org/). The three-dimensional structure of PPO1 was predicted by SWISS-MODEL (http://swissmodel.expasy.org/) and visualized using Swiss-PdbViewer v4.1.0 (http://www.expasy.org/spdbv/).

### 2.9 Statistical analysis

DNAMAN v5.1 (Lynnon Biosoft, Quebec, Canada) was used for sequence analysis. The statistical significance of differences in pairwise comparisons of transgenic lines and non-transformed control or mutants and wild types was assessed using Student’s *t*-test in Microsoft Excel 2016.

## 3 Results

### 3.1 Validation of the impact of *Ppo1* on PPO activity

Three non-segregant positive T_3_ transgenic lines (designated as 281-1A, 268-3A, and 235-1B, respectively), resistant to kanamycin and containing the RNAi cassette pRNAiPPO1, were obtained and characterized. The morphology and development characteristics showed no significant differences between transgenic plants and non-transformed controls. However, compared with non-transformed controls, the germination rate of transgenic plants was lower.

**Figure 2:**
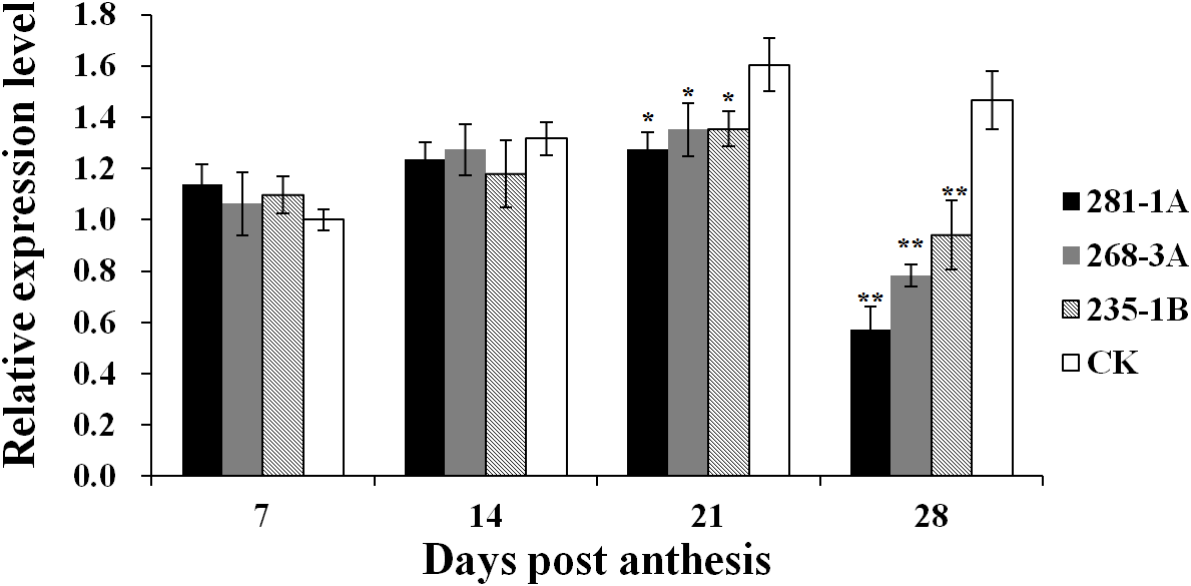
Expression levels of *Ppo1* in grains of T_3_ transgenic lines and non-transformed controls during different grain development stages. Gene expression levels were given as the expression levels relative to the values of the controls at 7 DPA (set to 1) after normalization to *β-actin* levels. Data were presented as means ± standard error from three biological replicates with three technical replicates each. Significant difference in transgenic lines compared with the controls is represented by one or two asterisks with the following significance levels: *P < 0.01, **P < 0.001. CK, non-transformed controls.

**Figure 3:**
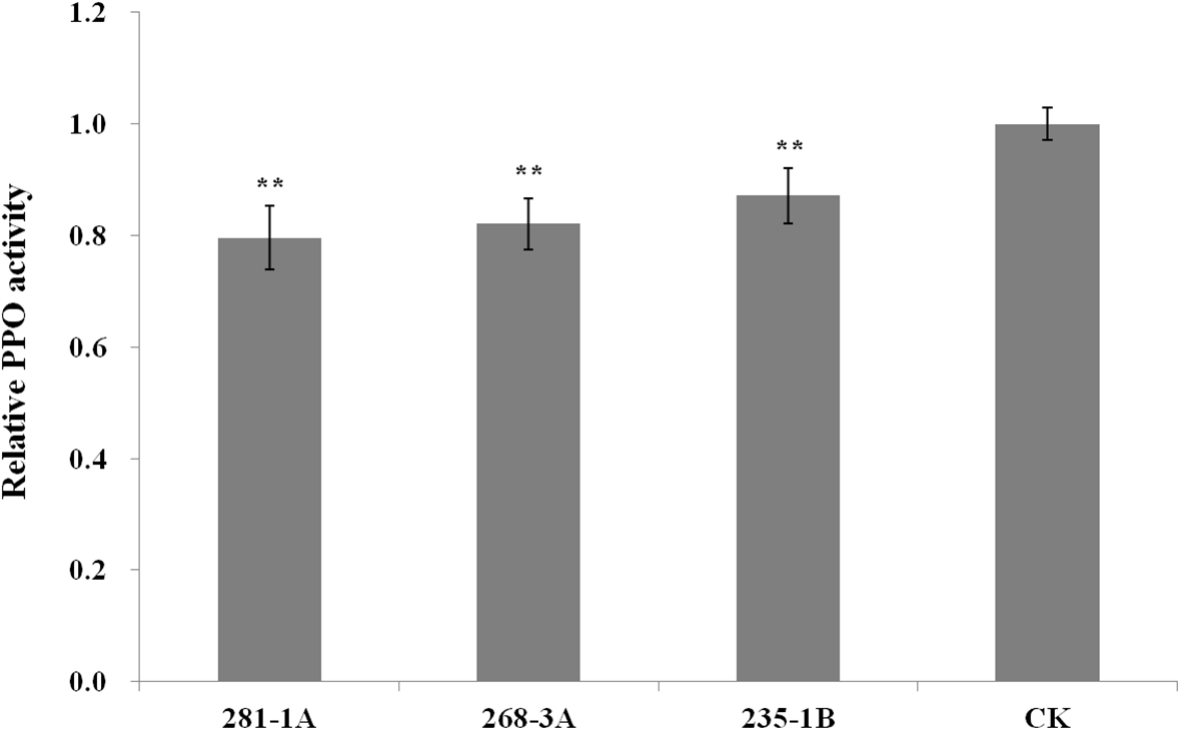
Relative PPO activity of T_3_ transgenic lines and non-transformed controls. Data were given as the fold relative to the values of non-transformed controls (set to 1). Five biological replicates were performed in triplicate and data were presented as means ± standard error. The double asterisk indicates significant differences between transgenic lines and controls at a *P* < 0.001 probability level. CK, non-transformed controls.

As shown in **Figure 2**, the transcript level of *Ppo1* showed no significant difference between transgenic lines and controls in the early stages of grain development (7 and 14 DPA). However, the transcript level of *Ppo1* of the transgenic lines was significantly reduced by 15.5%–20.4% and 35.8%–60.9% at 21 and 28 DPA, respectively. Accordingly, transgenic lines showed a significantly decreased PPO activity compared with non-transformed controls, ranging from 12.9% in the 235-1B line to 20.4% in the 281-1A line (**Figure 3**).

### 3.2 Subcellular localization of PPO1

**Figure 4:**
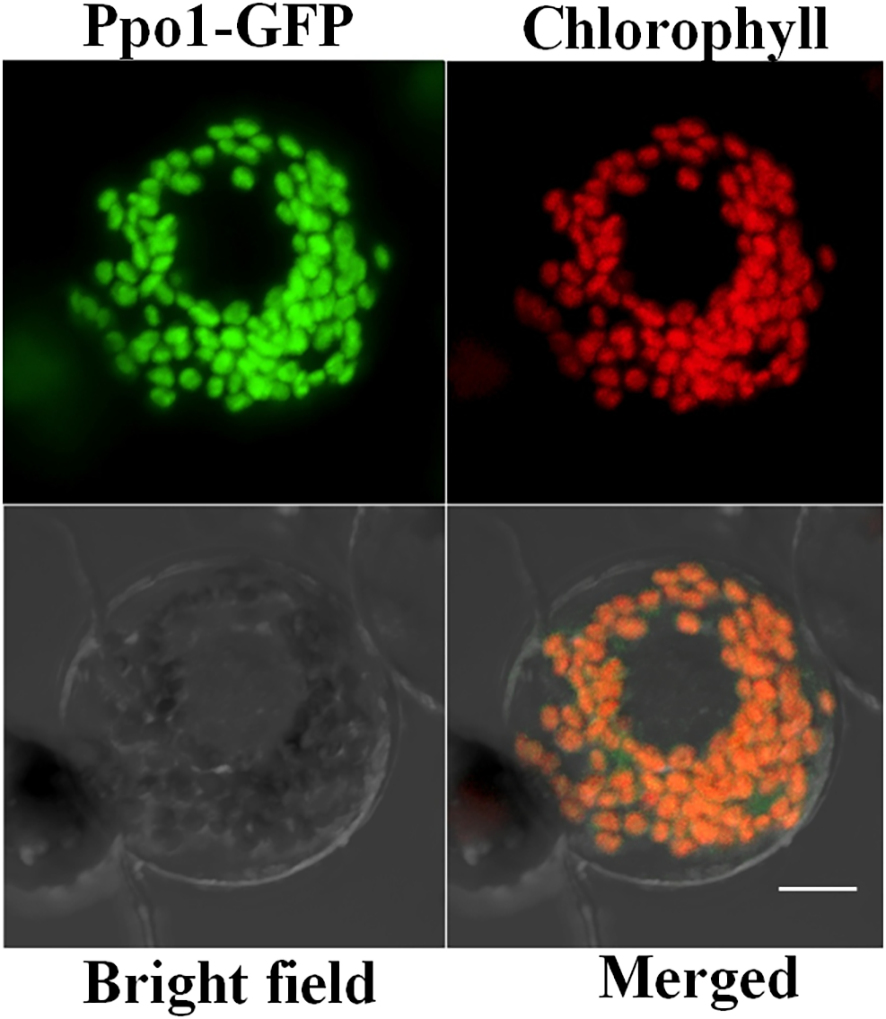
The confocal microscopy image indicating the subcellular localization of PPO1 in wheat protoplasts. Ppo1-GFP (green), chlorophyll autofluorescence (red), bright-field images, and an overlay of green and red signals are presented. Bar, 10 μm.

The subcellular location of PPO1 is very crucial for ascribing a physiological role to the enzyme. Ppo1-GFP was transiently expressed in wheat protoplasts to investigate PPO1 subcellular localization. As expected, GFP alone was distributed evenly in the cytoplasm and nuclei (data not shown), whereas the Ppo1-GFP fusion proteins co-localized exclusively with the autofluorescent signals of chlorophyll in the chloroplasts characterized by a small disc pattern, indicating that PPO1 was localized in plastids (**Figure 4**).

### 3.3 Screening of *Ppo1* mutants

A total of 32 *Ppo1* mutants were detected in 2,491 M_2_ EMS-mutagenized populations by TILLING (**Table S5**). Sequence analysis showed that the frequency of mutation from C to T was 43.8% and that from G to A was 53.1%. In addition, a specific mutation type from A to G was detected in M091139. Classified by mutation position, eight mutations were distributed in the intron region and 24 mutations were located in the exon region, and the latter were further divided into eight missense mutations and 16 synonymous mutations. Due to the non-denatured PAGE used to separate CEL I digestion products, the mismatch within 150 bp of the amplified fragment exceeded the detection range and could not be detected. Therefore, the mutation density of the *Ppo1* gene in these EMS-mutagenized populations was 1/187.5 kb.

### 3.4 Effect of mutation sites on PPO1 function

**Figure 5:**
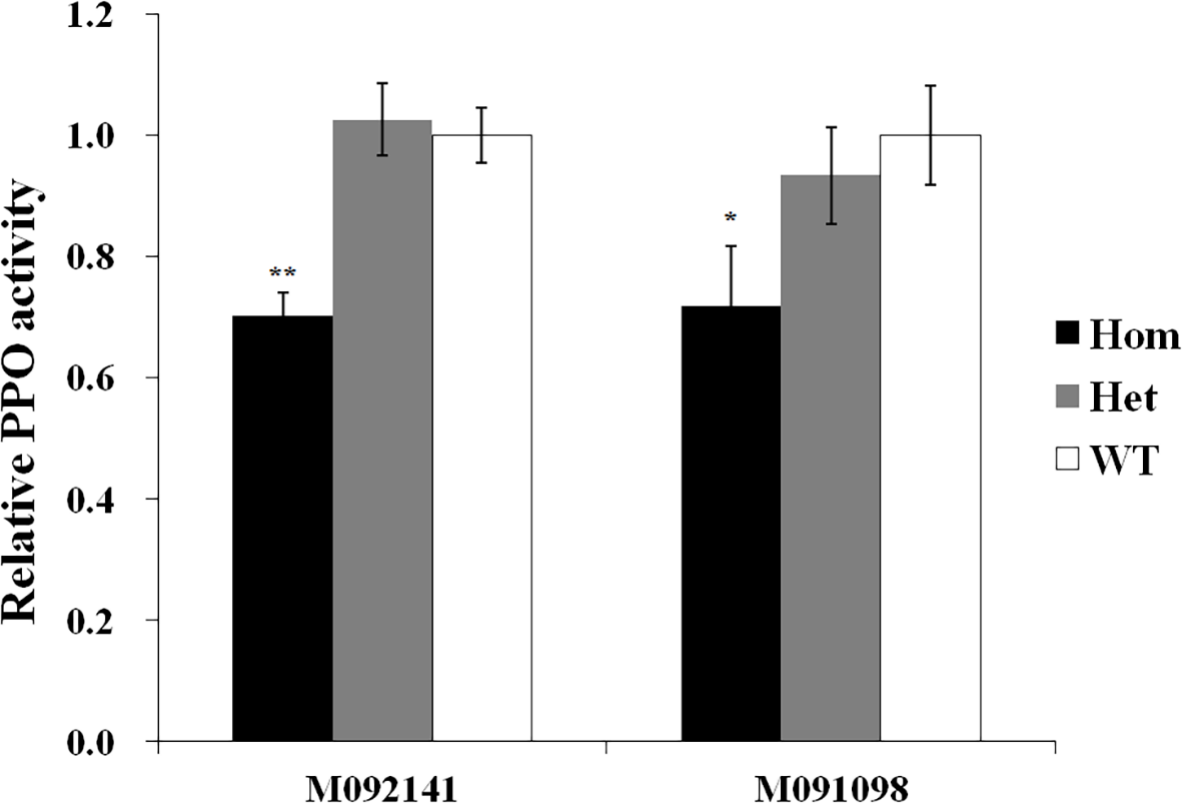
Relative PPO activity of different genotypes in F_2_ populations derived from homozygous missense mutants crossed with the respective control plants. Data were given as the fold relative to the values of wild-type plants (set to 1) in each F_2_ population. Five biological replicates were performed and the data were presented as means ± standard error. Significant difference between homozygous or heterozygous mutant plants and wild-type plants in each F_2_ population is represented by one or two asterisks with the following significance levels: *P < 0.01, **P < 0.001. Hom, homozygous mutant; Het, heterozygous mutant; WT, wild-type plants.

Among eight F_2_ populations developed from homozygous missense mutants backcrossed with wild-type plants, only in the F_2_ populations from mutants M092141 and M091098 the PPO activity of homozygous mutants was significantly lower than that of wild type, decreased by 29.7% and 28.8%, respectively, whereas there was no significant difference of PPO activity between heterozygous mutants and wild-type plants (**Figure 5**).

**Figure 6:**
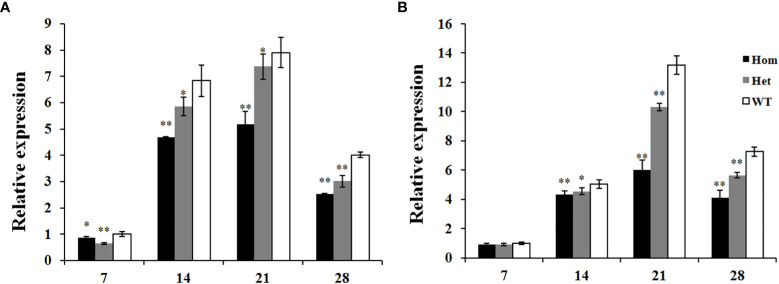
The expression analysis of the *Ppo1* gene in grains of three genotypes in each F_2_ population during different grain development stages. **(A)** M092141, **(B)** M091098. The transcript levels of these genotypes were given as the expression levels relative to the values of wild-type plants at 7 DPA (set to 1) after normalization to *β-actin* levels. Data were presented as means ± standard error from five biological replicates. Significant difference between homozygous or heterozygous mutant plants and wild-type plants in each F_2_ population is represented by one or two asterisks with the following significance levels: *P < 0.05, **P < 0.01. Hom, homozygous mutant; Het, heterozygous mutant; WT, wild-type plants.

The expression profiles of *Ppo1* in these two F_2_ populations were further analyzed. In the F_2_ population constructed by the M092141 mutant, the total expression of the *Ppo1* gene of homozygous and heterozygous mutants was significantly lower than that of the wild-type plants at all stages of grain development, decreased by 14.1%–37.1% and 6.7%–35.9%, respectively (**Figure 6A**). In the F_2_ population constructed by the M091098 mutant, the total expression of the *Ppo1* gene of homozygous and heterozygous mutants was significantly lower than that of the wild-type plants at 14–28 DPA, decreased by 14.3%–54.4% and 10.1%–22.4%, respectively, while there was no significant difference of *Ppo1* gene expression among genotypes at 7 DPA (**Figure 6B**).

### 3.5 Functional domain and structure of PPO1

Using NCBI’CDD, three functional domains were identified in PPO1 protein, namely, tyrosinase (pfam00264, positions 158–367), PPO1_DWL (pfam12142, positions 373–423), and PPO1_KFDV (pfam12143, positions 440–575) (**Figure S1**). Based on the *Ppo1* amino acid sequences of 74 plant species such as *Arabidopsis thaliana*, *Oryza sativa*, and *Hordeum vulgare* (**Table S4**), five conserved domains were detected using MEME Suite v5.1.0 (**Figure S1**). The missense mutation sites of M090168 (E144K), M092141 (G311S), M091098 (G299R), and M092466 (D413N) were just located within domain 1, domain 3 (2), and domain 5, respectively. Unlike the mutation sites of M092141 and M091098 as mentioned above, which significantly decreased PPO activity and *Ppo1* expression level (**Figures 5**, **6**), there was no significant difference in PPO activities and *Ppo1* expression levels among homozygous, heterozygous, and wild-type genotypes in the F_2_ populations from mutants M090168 and M092466 (**Figures S2**, **S3**).

Sequence alignments showed that mutation sites of M092141 and M091098 were highly conserved, where the frequency of glycine (G) in these 74 plant species was 94.59% and 100%, respectively, and serine (S) and arginine (R) were not present (**Table 1**). For the mutation site of M090168, the frequency of glutamic acid (E) and lysine (K) was almost identical in these 74 plant species, displaying 24.32% and 20.27%, respectively. For the mutation site of M092466, the frequency of aspartic acid (D) was 67.57%, and the frequency of asparagine (N) was 5.41%.

**Table 1:** The amino acid sequences at mutation sites located in the conserved domains among 74 plant species.

Site^a^	Amino acid (frequency in 74 plant species, %)								
144	E (24.32)	K (20.27)	N (18.92)	S (17.57)	A (6.76)	T (5.41)	Q (4.05)	M (1.35)	R (1.35)
299	G (100)								
311	G (94.59)	A (2.70)	R (1.35)	N (1.35)					
413	D (67.57)	E (9.46)	N (5.41)	K (4.05)	Q (4.05)	G (2.70)	S (2.70)	P (2.70)	T (1.35)

^a^ The number is its position from methionine in PPO1.

**Figure 7:**
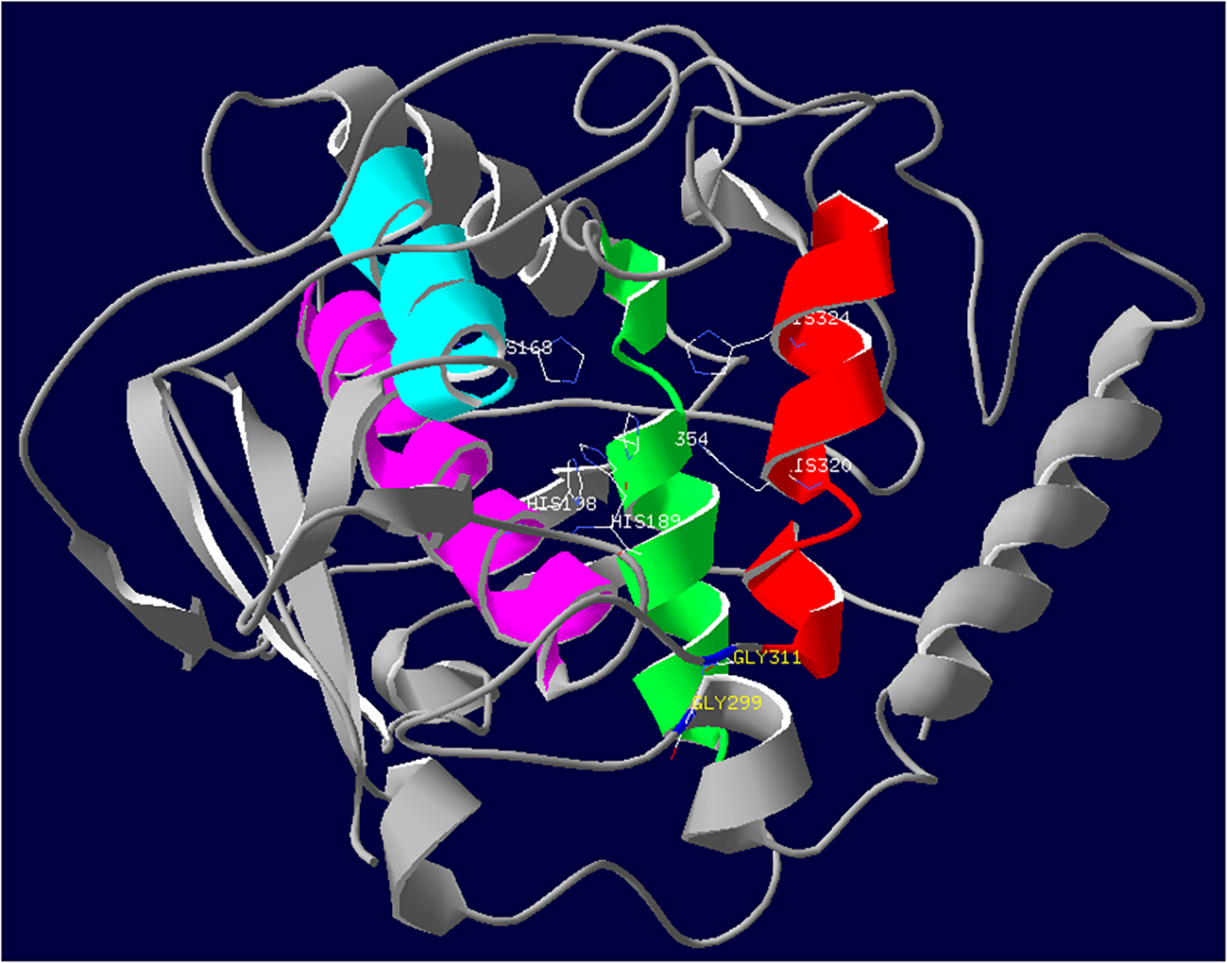
Three-dimensional structure of PPO1. The active site of PPO1 containing a binuclear copper center is located in a four-helix bundle and shown in color (red, green, blue, and purple). Side chains of six histidine residues (His 168, His 189, and His 198; His 320, His 324, and His 354) coordinating with two copper ions are colored by atom type (carbon is white; nitrogen is blue). Mutation sites of the two missense mutants M092141 (G311S) and M091098 (G299R) that significantly reduced *Ppo1* gene expression and PPO activity are displayed in blue.

In the three-dimensional structure model, the active site of PPO1 showed a hydrophobic pocket formed by a four-α-helix bundle, which encloses two copper-binding regions (**Figure 7**). Each copper ion was coordinated by three histidine (His) residues. The CuA was coordinated by His 168, His 189, and His 198. Similarly, the CuB was coordinated by His 320, His 324, and His 354. The mutation sites of M092141 and M091098 were adjacent to the entrance of the hydrophobic pocket of the active site.

## 4 Discussion

### 4.1 *Ppo1-*specific silencing and function verification

RNAi is widely accepted as an approach having great potential to analyze gene function and is particularly useful in polyploidy plants, such as wheat, because all homoeologous copies could be simultaneously silenced with a single RNAi construct (Travella et al., 2006; Fu et al., 2007). It has been suggested that 88%–100% nucleotide identity and the presence of a continuous stretch of at least 21 identical nucleotides between the trigger fragment and target genes are crucial for a successful gene silencing in higher plants (Wesley et al., 2001; Fu et al., 2007; McGinnis et al., 2007).

In the present study, a part of the third exon of *Ppo-A1* (454 bp) was selected as the trigger fragment, which shares 94.1% and 97.2% nucleotide similarity with *Ppo-B1* and *Ppo-D1*, respectively. In addition, there also exist eight contiguous stretches of identical nucleotides longer than 21 nt, ranging from 25 to 54 nt. As expected, compared with the control, the level of *Ppo1* transcript in RNAi transgenic lines was drastically decreased by 15.5%–60.9%, and PPO activity was significantly reduced by 12.9%–20.4%, confirming the important role of *Ppo1* on grain PPO activity (**Figures 2**, **3**). Notably, the transcript level of *Ppo1* in transgenic lines was significantly reduced at the later stages of the grain development period (21 and 28 DPA), with the peak decreasing at 28 DPA (**Figure 2**). This might be due to the use of a promoter of *Bx17* in the RNAi cassette pRNAiPPO1 since the HMW-GS is usually expressed in the middle and late stages of grain development (Shewry et al., 2009). Meanwhile, the expression level of *Ppo1* was higher at later development stages (**Figure 2**).

Recently, a new paralogous *Ppo2* gene family also was mapped on homoeologous group 2 chromosomes (Beecher and Skinner, 2011; Beecher et al., 2012; Taranto et al., 2015). Three homoeologs of *Ppo2* share 86.4%–86.7% nucleotide similarity with *Ppo-A1* within the 460-bp trigger fragment. There were only three contiguous stretches of identical nucleotides over 21 nt. qPCR analysis also indicated that there was no significant difference in *Ppo2* expression between transgenic lines and controls (**Figure S4**). Therefore, we inferred that the pRNAiPPO1 specifically silenced *Ppo1* expression rather than *Ppo2*, and the function of *Ppo2* on PPO activity will be investigated in further study.

### 4.2 Genetic regulation of PPO1 function

TILLING is an efficient method to explore gene function and regulation, producing large series of mutated alleles that may affect protein function and generate partial phenotypic changes or intermediate expression of target genes (Chen et al., 2014; Irshad et al., 2020). A total of 32 mutations of *Ppo1* genes were detected in the present study, providing important resources for gene functional analysis and investigating the regulation of amino acids or regions on PPO1 function.

The active site of PPO exhibiting a hydrophobic pocket consists of two copper-binding active regions (CuA and CuB) (Klabunde et al., 1998; Gerdemann et al., 2002; Jukanti, 2017). Out of eight missense mutants, mutation sites of M092141 and M091098 significantly reduced the expression of *Ppo1* genes by 6.7%–37.1% and 10.1%–54.4%, accompanied by a decreased PPO activity (29.7% and 28.8%), respectively (**Figures 5**, **6**). The three-dimensional structure showed that mutation sites of M092141 (G311S) and M091098 (G299R) were adjacent to the entrance of the pocket of the active site. When glycine mutates to serine or arginine, the longer side chains may hinder substrate binding. Meanwhile, these mutation sites were well conserved among known *Ppo1* genes in 74 plant species, where the frequency of glycine was 94.6% and 100%, respectively (**Table 1**). All these indicated that the two mutation sites were very important for PPO1 function, which affected PPO activity by decreasing gene expression or interfering with enzyme–substrate affinity. Notably, in the F_2_ populations constructed by M092141 and M091098, the *Ppo1* expression levels of heterozygous mutants were significantly lower than those of wild-type plants (**Figure 6**), while there was no significant difference in PPO activities between them (**Figure 5**). We inferred that the effect of reduced *Ppo1* expression on PPO activity might be compensated by post-transcription, translation, or post-translation regulation in these heterozygous F_2_ individuals.

### 4.3 PPO activity affecting wheat germination

The mutation density of *Ppo1* genes in the EMS mutagenesis population was estimated to be 1/187.5 kb in this study. This mutation frequency was much lower than that in hexaploid wheat detected by Uauy et al. (2009) using the non-denaturing PAGE technology. This discrepancy may be attributed to the different mutant populations, the different G/C contents in the gene target region, or the low detection sensitivity of the non-denaturing PAGE for the mixed pool of eight samples. Notably, the germination rate of RNAi transgenic plants became lower compared with non-transformed controls, while the germination rate of transgenic plants returned to the normal level when introducing PPO artificially (Aladdin Industrial Corp., data not shown). Previous studies also identified that PPO is active during wheat seed germination (Demeke et al., 2001b; Ölçer and Mecit, 2012). Therefore, we inferred that a low level of PPO activity in grain may affect wheat germination. Mutants may not be germinated normally if their mutation sites seriously influence PPO1 function and PPO activity, which results in fewer mutants being germinated, survived, and screened out. Of course, more investigations are needed to substantiate this hypothesis.

### 4.4 Superior germplasm for quality improvement

Color is an important criterion for the quality of wheat products. The browning during processing and storage not only affects the apparent color of flour products but also negatively affects their flavor, nutritional properties, and shelf life. PPO has been implicated as a main factor contributing to the darkening and discoloration of wheat-based end products (Feillet et al., 2000; Anderson et al., 2006). Therefore, huge efforts have been made to develop bread wheat varieties with low PPO activity in grains to reduce the time-dependent darkening (Wang et al., 2009; Martin et al., 2011; Talbert et al., 2013). However, the lack of a comprehensive molecular mechanism of PPO activity and superior germplasm resources seriously affects the breeding process. Common wheat still shows a considerably high PPO activity in comparison with durum wheat cultivars that generally have very low to almost nil PPO activity (Asenstorfer et al., 2014; Salaria et al., 2018).

Mutants identified by TILLING can be readily used in traditional breeding programs since the technology is non-transgenic. In this study, M092141 and M091098 significantly reduced grain PPO activity, while they have no significant difference in agronomic characters compared with the wild type. Notably, these two mutants were derived from the leading wheat cultivar Jimai 22 which is a high-yield and widely adaptable wheat cultivar and grown in an average area of over 1.4 million ha annually during the past 16 years in China. Therefore, they can be used as important germplasm for breeding wheat varieties with low PPO activity without further pre-breeding to remove undesirable agronomic traits.

## 5 Conclusions

PPO activity in wheat grain is the main cause of the browning of flour products, resulting in considerable economic and nutritional losses. Characterization of the molecular mechanism of PPO activity is an important prerequisite for developing wheat cultivars with low grain PPO activity. In this study, the effect of *Ppo1* on grain PPO activity was verified, where the RNAi-mediated downregulation of *Ppo1* expression resulted in a remarkable reduction in PPO activity. A total of 32 mutants of *Ppo1* genes were identified by TILLING, providing important resources for the analysis of gene function and genetic regulation. It was found that conserved amino acids (G299 and G311) adjacent to the entrance of the pocket of the active site of PPO1 may affect gene function by changing gene expression or interfering with enzyme–substrate affinity. M092141 and M091098 significantly decreased *Ppo1* expression level and PPO activity in grain and had no negative effects on agronomic traits; two mutants can be used as donor parents for breeding wheat varieties with low grain PPO activity to reduce the browning of flour-based products. This study would facilitate further understanding of the molecular mechanism of grain PPO activity and broaden genetic resources for wheat quality improvement.

## Data availability statement

The datasets presented in this study can be found in online repositories. The names of the repository/repositories and accession number(s) can be found in the article/**Supplementary Material**.

## Author contributions

SZ performed the experiment and wrote the paper. HL and HC analyzed the data. HSL, CL, JS, and AL participated in the field trials. WW and JZ assisted in writing the paper. ZH, XX, and JL designed the experiment and wrote the paper. All authors read and approved the final manuscript.
