# Supplementary material for: Functional analysis of polyphenol oxidase 1 gene in common wheat
Source: Front Plant Sci. 2023 Jul 31;14:1171839. doi: 10.3389/fpls.2023.1171839 (PMC10424926; doi:10.3389/fpls.2023.1171839)
Supplement: Supplementary file 1 [file DataSheet_1.docx]

Supplementary Material

Functional analysis of polyphenol oxidase 1 gene in common wheat

Shengnan Zhai^1^, Hang Liu^2^, Xianchun Xia^3^, Haosheng Li^1^, Xinyou Cao^1^, Zhonghu He^3^, Wujun Ma^2^, Cheng Liu^1^, Jianmin Song^1^, Aifeng Liu^1^, Jingjuan Zhang^2^, Jianjun Liu^1^*

*** Correspondence:** Jianjun Liu: [ljjsaas@163.com](mailto:ljjsaas@163.com)


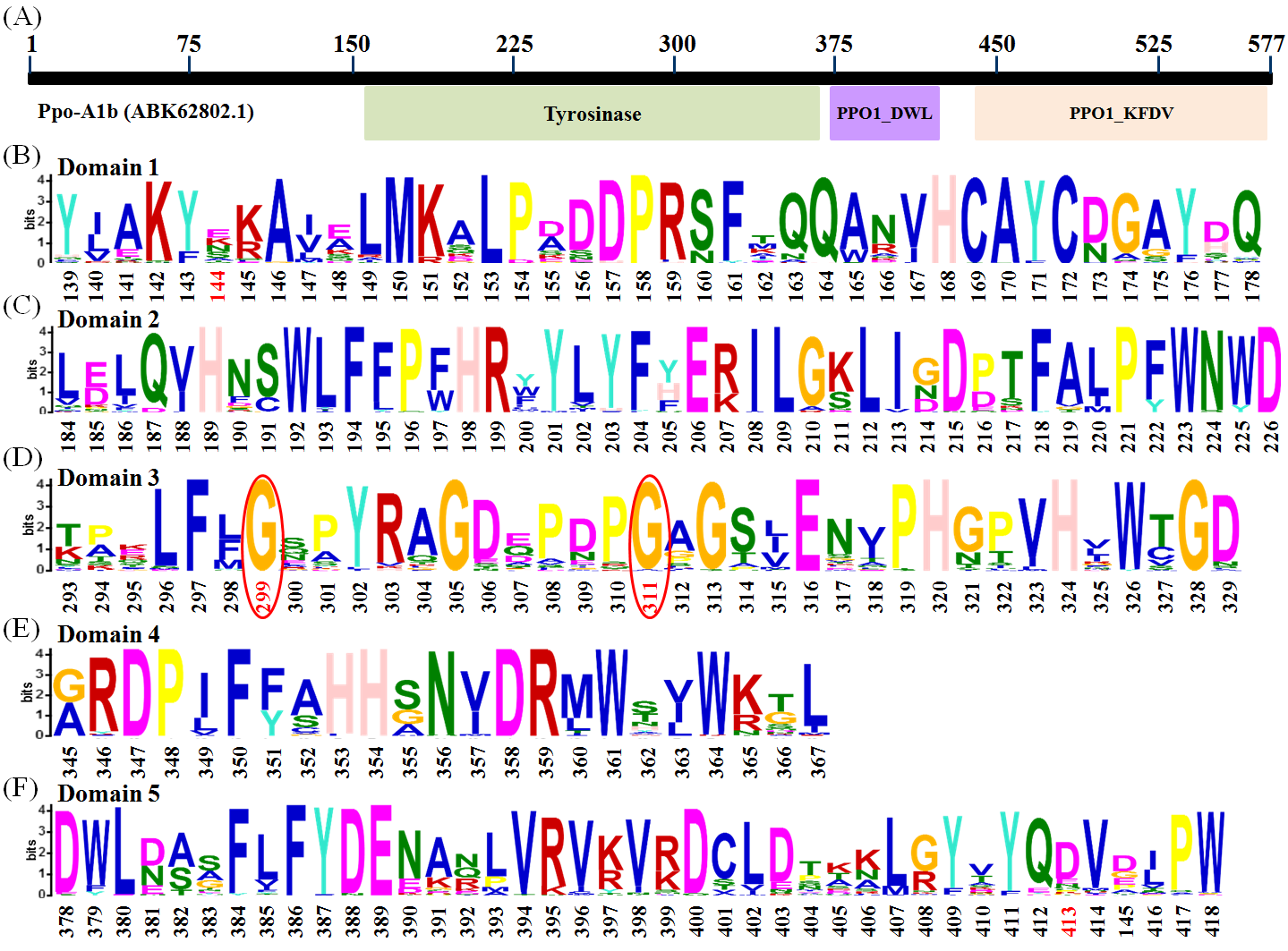


**Supplementary Figure 1** The prediction of functional domains of PPO1. (A) three functional domains identified by NCBI’CDD. (B)-(F) five conserved domains detected using MEME Suite v5.1.0 based on known *Ppo1* amino acid sequences in 74 plant species. The number is its position from smethionine in PPO1. Mutation sites of missense mutants located in the conserved domains are displayed in red. Two missense mutations M092141 (G311S) and M091098 (G299R) significantly reduced *Ppo1* gene expression and PPO activity is circled in red.

**Supplementary Figure 2** Relative PPO activity of different genotypes in F_2_ populations derived from homozygous missense mutants M090168 and M092466 crossed with respective control plants. Data were given as the fold relative to the values of wild-type plants (set to 1) in each F_2_ population. Five biological replicates were performed and the data were presented as means ± standard error.

**Supplementary Figure 3** The expression analysis of *Ppo1* gene in grains of three genotypes in each F_2_ population during different grain development stages*.* (A) M090168, (B) M092466. Transcript levels of these genotypes were given as the expression levels relative to the values of wild-type plants at 7 DPA (set to 1) after normalization to *β-actin* levels. Data were presented as means ± standard error from five biological replicates.

**Supplementary Figure 4** Expression levels of *Ppo2* in grains of T_3_ transgenic lines and non-transformed controls during different grain development stages. Gene expression levels were given as the expression levels relative to the values of the controls at 7 DPA (set to 1) after normalization to *β-actin* levels. Data were presented as means ± standard error from three biological replicates with three technical replicates each.

**Supplementary Table 1** Primers used for the RNAi vector construction and positive transgenic lines detection

| Name | Sequence (5’-3’) |
| --- | --- |
| PPOBamHIF | AATTGGATCCGTACCGGTGGTGGCGGG |
| PPOAsuIIR | GACGTTCGAATGATTTCGAACGCGAAGAAG |
| PPOKpnIF | AGGGTGGCAGGGAGGGTA |
| PPONheIR | GAGTGCTAGCTGATTTCGAACGCGAAGAAG |
| In-F | GCGAGGTACCGTAAGCCACTCACTCACTACCAATAC |
| In-R | GCGCGGATCCCTGGGAAATTATTCGAAACACC |
| FAD-F | ATTCTTATTTCTTTCCAGTAGC |
| FAD-R | AGAAGCGGCATAATGTGAGA |

**Supplementary Table 2** Primers developed for qRT-PCR analysis

| Gene | Name | Sequence (5’-3’) |
| --- | --- | --- |
| *Ppo1* | Ppo1-F | GAGAGGGCCGTGGCGCTCATGA |
|  | Ppo1-R | GAGGTAGAACCTGTGCCATGGG |
| *β-actin* | Actin-F | CTGATCGCATGAGCAAAGAG |
|  | Actin-R | CCACCGATCCAGACACTGTA |
| *Ppo2* | *Ppo2-F* | GCCGCCGTTCCCGGTCGACCTC |
|  | *Ppo2-R* | CACYCGACACCATCTGGCGGTA |

**Supplementary Table 3** Primers developed for identifying mutations in *Ppo1* by TILLING

| Gene | Name | Sequence (5’-3’) | Length (bp) |
| --- | --- | --- | --- |
| *Ppo-A1b* | A1F | ACCTTCTCCGTGGCCTTCACCT | 1037 (74-1110) |
|  | A2R | CTCTTGGGATATTGGTGTCGGA |  |
| *Ppo-A1b* | A4F | CGTATGGTCAATGGGTTATGGA | 1035 (791-1825) |
|  | A10R | CACCTCCTCCTCCTCGTCCTTT |  |
| *Ppo-B1a* | B6F | CGCCAGGCCAGTAGTACTATAT | 956 (955-1910) |
|  | B17R | GAAATGGTCGGCGATCTCGATT |  |
| *Ppo-D1a^a^* | PPO16F | TGCTGACCGACCTTGACTCC | 713 (873-1585) |
|  | PPO16R | CTCGTCACCGTCACCCGTAT |  |
| *Ppo-D1b^b^* | PPO29F | TGAAGCTGCCGGTCATCTAC | 490 (798-1287) |
|  | PPO29R | AAGTTGCCCATGTCCTCGCC |  |

^a^ Jimai20 mutant screening

^b^ Jimai22 mutant screening

**Supplementary Table 4** The information of known *Ppo1* genes in 74 plant species

| No. | Organism | Gene ID |
| --- | --- | --- |
| 1 | *Eucalyptus grandis* | Eucgr.D00657 |
| 2 | *Musa acuminata* | GSMUA_Achr6G29370_001 |
| 3 | *Miscanthus sinensis* | Misin11G197000 |
| 4 | *Oropetium thomaeum* | Oropetium_20150105_01740 |
| 5 | *Oryza sativa* | OsKitaake04g268200 |
| 6 | *Panicum hallii* | PhHAL.7G286100 |
| 7 | *Setaria viridis* | Sevir.7G252200 |
| 8 | *Pharus latifolius* | Phala.06G236300 |
| 9 | *Sorghum bicolor* | SbiSC187.06G176700 |
| 10 | *Zea mays* | ZmPHB47.10G202800 |
| 11 | *Trifolium pratense* | Tp57577_TGAC_v2_gene14726 |
| 12 | *Urochloa fusca* | Urofu.7G280200 |
| 13 | *Thinopyrum intermedium* | Thint.06G0534700 |
| 14 | *Anacardium occidentale* | Anaoc.1140s0003 |
| 15 | *Carica papaya* | evm.TU.supercontig_530.1 |
| 16 | *Eleusine coracana* | ELECO.r07.4AG0303420 |
| 17 | *Chasmanthium laxum* | Chala.06G241800 |
| 18 | *Daucus carota* | DCAR_019634 |
| 19 | *Hordeum vulgare* | HORVU.MOREX.r3.2HG0194690 |
| 20 | *Ananas comosus* | Aco014847 |
| 21 | *Arachis hypogaea* | arahy.Tifrunner.gnm1.ann1.EW9UD1 |
| 22 | *Lotus japonicus* | Lj6g0022823 |
| 23 | *Lupinus albus* | Lalb_Chr20g0122021 |
| 24 | *Glycine soja* | GlysoPI483463.13G200100 |
| 25 | *Medicago truncatula* | Medtr2g013010 |
| 26 | *Spirodela polyrhiza* | Spipo24G0019000 |
| 27 | *Vigna unguiculata* | Vigun06g204500 |
| 28 | *Vitis vinifera* | VIT_200s0480g00060 |
| 29 | *Betula platyphylla* | BPChr03G28912 |
| 30 | *Brachypodium distachyon* | Bradi2g52210 |
| 31 | *Carya illinoinensis* | CiPaw.09G054600 |
| 32 | *Corymbia citriodora* | Cocit.D0445 |
| 33 | *Cicer arietinum* | Ca_00278 |
| 34 | *Lindenbergia philippensis* | Liphi.06G169500 |
| 35 | *Marchantia polymorpha* | Mapoly0117s0058 |
| 36 | *Joinvillea ascendens* | Joasc.12G064500 |
| 37 | *Populus trichocarpa* | PtStettler14.01G343700 |
| 38 | *Salix purpurea* | Sapur.016G249400 |
| 39 | *Vaccinium darrowii* | Vadar_g12823 |
| 40 | *Hydrangea quercifolia* | Hyque.01G009700 |
| 41 | *Castanea dentata* | Caden.02G251000 |
| 42 | *Citrus clementina* | Ciclev10014693m.g |
| 43 | *Malus domestica* | MD10G1298500 |
| 44 | *Poncirus trifoliata* | Ptrif.0002s2716 |
| 45 | *Portulaca amilis* | FUN_008721 |
| 46 | *Quercus rubra* | Qurub.10G027900 |
| 47 | *Chenopodium quinoa* | AUR62016960 |
| 48 | *Dioscorea alata* | Dioal.15G106400 |
| 49 | *Fragaria vesca* | gene30434-v1.0-hybrid |
| 50 | *Triticum aestivum* | TraesCS2A02G468200.1 |
| 51 | *Phaseolus lunatus* | Pl02G0000355600.v1 |
| 52 | *Sphagnum magellanicum* | Sphmag12G108700 |
| 53 | *Coffea arabica* | evm.TU.Scaffold_618.894 |
| 54 | *Zostera marina* | Zosma05g20900 |
| 55 | *Lactuca sativa* | Lsat_1_v5_gn_9_102220 |
| 56 | *Kalanchoe laxiflora* | Kalax.0092s0048 |
| 57 | *Olea europaea* | Oeu009882.1 |
| 58 | *Cinnamomum kanehirae* | CKAN_01745500 |
| 59 | *Beta vulgaris* | EL10Ac8g20317 |
| 60 | *Cucumis sativus* | Cucsa.126490 |
| 61 | *Theobroma cacao* | Thecc.06G192400 |
| 62 | *Paspalum vaginatum* | Pavag07G073400 |
| 63 | *Spinacia oleracea* | Spov3_chr2.04266 |
| 64 | *Thuja plicata* | Thupl.29377680s0007 |
| 65 | *Gossypium darwinii* | Godar.D09G095700 |
| 66 | *Amaranthus hypochondriacus* | AH021624 |
| 67 | *Solanum tuberosum* | PGSC0003DMG400018913 |
| 68 | *Acorus americanus* | Acora.08G131900 |
| 69 | *Linum usitatissimum* | Lus10024122.g |
| 70 | *Helianthus annuus* | HanXRQChr15g0492231 |
| 71 | *Aquilegia coerulea* | Aqcoe1G318200 |
| 72 | *Prunus persica* | Prupe.4G041400 |
| 73 | *Manihot esculenta* | Manes.17G042600 |
| 74 | *Mimulus guttatus* | MgNONTOL.M1195 |

**Supplementary Table 5** Summary of mutations in *Ppo1* identified by TILLING

| Gene | M_3_ Plant | Variety | Exon/ | Nucleotide  change^a^ | Codon  change | Amino acid  change^b^ | Zygosity^c^ |
| --- | --- | --- | --- | --- | --- | --- | --- |
|  |  |  | Intron |  |  |  |  |
| *Ppo-A1* | M091139 | J22 | Intron | A857G |  |  | Hom |
|  | M091507 | J22 | Exon | C1045T | CCC→CTC | P251L | Het |
|  | M091352 | J22 | Exon | C1090T | TCC→TTC | S266F | Hom |
|  | M091655 | J22 | Exon | C1094T | GAC→GAT | D267= | Het |
|  | M092204 | J20 | Exon | C1145T | TAC→TAT | Y284= | Hom |
|  | M091430 | J22 | Intron | C1255T |  |  | Hom |
|  | M091977 | J22 | Exon | C1337T | GAC→GAT | D306= | Het |
|  | M091653 | J22 | Exon | C1444T | TTC→TTT | F342= | Hom |
|  | M090896 | J20 | Exon | C1469T | TTC→TTT | F350= | Hom |
|  | M090439 | J20 | Exon | C333T | GGC→GGT | G111= | Hom |
|  | M090485 | J20 | Intron | C856T |  |  | Hom |
|  | M092569 | J20 | Exon | C956T | CCC→CCT | P221= | Hom |
|  | M091125 | J22 | Exon | G1108A | AGA→AAA | R272K | Hom |
|  | M090561 | J20 | Exon | G1294A | AAG→AAA | K292= | Hom |
|  | M090663 | J20 | Exon | G1385A | ACG→ACA | T322= | Hom |
|  | M090752 | J20 | Exon | G411A | GCG→GCA | A137= | Hom |
|  | M090168 | J20 | Exon | G430A | GAG→AAG | E144K | Hom |
|  | M092627 | J20 | Exon | G486A | GAG→GAA | E162= | Hom |
|  | M091206 | J22 | Exon | G561A | CAG→CAA | Q187= | Hom |
|  | M092434 | J20 | Intron | G717A |  |  | Hom |
|  | M092270 | J20 | Intron | G848A |  |  | Hom |
|  | M090557 | J20 | Intron | G873A |  |  | Het |
|  | M090886 | J20 | Intron | G886A&G1241A |  |  | Hom |
| *Ppo-B1* | **M092141** | **J22** | **Exon** | **G1398A** | **GGC→AGC** | **G311S** | **Het** |
| *Ppo-D1* | M092728 | J20 | Intron | 1068AA |  |  | Hom |
|  | M090292 | J20 | Exon | C1187T | CAC→CAT | H320= | Hom |
|  | M090413 | J20 | Exon | C1382T | CTC→CTT | L385= | Hom |
|  | M090267 | J20 | Exon | C1473T | CTG→TTG | L416= | Het |
|  | M091187 | J22 | Exon | G1159A | CTG→CTA | L298= | Hom |
|  | **M091098** | **J22** | **Exon** | **G1160A** | **GGA→AGA** | **G299R** | **Het** |
|  | M092444 | J20 | Exon | G1242A | GGC→AGC | G339S | Het |
|  | M092466 | J20 | Exon | G1464A | GAC→AAC | D413N | Hom |

^a^ the first letter indicates the wild type nucleotide, the number is its position from the start codon, and the last letter is the mutant nucleotide;

^b^ the first letter indicates the wild type amino acid, the number is its position from the smethionine, and the last letter is the mutant amino acid; =, synonymous mutation;

^c^ Hom, homozygous genotype; Het, heterozygous genotype;

bold items indicate that mutations severely affect phenotype.
